# Rural Ambulatory Access for Semi-Urgent Care and the Relationship of Distance to an Emergency Department

**DOI:** 10.5811/westjem.2015.4.25485

**Published:** 2015-06-24

**Authors:** Ashley Parks, Andy Hoegh, Damon Kuehl

**Affiliations:** * Virginia Tech Carilion School of Medicine and Research Institute, Roanoke, Virginia; † Virginia Tech, Department of Statistics, Blacksburg, Virginia; ‡ Virginia Tech Carilion School of Medicine and Research Institute, Department of Emergency Medicine, Roanoke, Virginia

## Abstract

**Introduction:**

Availability of timely access to ambulatory care for semi-urgent medical concerns in rural and suburban locales is unknown. Further distance to an emergency department (ED) may require rural clinics to serve as surrogate EDs in their region, and make it more likely for these clinics to offer timely appointments. We determined the availability of urgent (within 48 hours) access to ambulatory care for non-established visiting patients, and assessed the effect of insurance and ability to pay cash on a patient’s success in scheduling an appointment in rural and suburban Eastern United States. We also assessed how proximity to EDs and urgent care (UC) facilities influenced access to semi-urgent ambulatory appointments at primary care facilities.

**Methods:**

The Appalachian Trail, which runs from Georgia to Maine, was used as a transect to select 190 rural and suburban primary care clinics located along its entire length. We calculated their location and distance to the nearest hospital-based ED or UC via Google Earth. A sham patient representing a non-established visiting patient called each clinic over a four-month period (2013), requesting an appointment in the next 48 hours for one of three scripted clinical vignettes representing common semi-urgent ambulatory concerns. We randomized the scenarios and insurance statuses (insured vs. uninsured). Each clinic was contacted twice, once with the caller representing an insured patient, once with the caller representing an uninsured patient. When the caller was representing an uninsured patient, any required upfront payment was requested from each clinic. One hundred dollars was used as a cutoff between the uninsured as a distinction between those able to afford substantial upfront sums and those who could not. To determine if proximity to other sources of care impacted a clinic’s ability to grant an appointment, distance to the nearest ED or UC was modeled as a dichotomous variable using 30 miles as the divider.

**Results:**

Of 380 requests, 96 (25.3%) resulted in appointments within 48 hours. Insured patients and uninsured patients able to pay a substantial amount upfront (>$100) were more likely to book an appointment (p-value <0.001, OR 18, CI [5–154]). Of the 47 clinics that granted uninsured patients appointments 89.3% required some form of payment up front. Farther distances from an ED did not result in greater likelihood of an appointment (OR 1.7, CI [0.4–11.3]). Clinics located within 30 miles of an UC were more likely to grant an appointment (OR 2.45, CI [1.19–5.80]).

**Conclusion:**

Almost 75% of rural clinics were unable to grant a new appointment for a semi-urgent health complaint. Lack of insurance and large upfront charges appear to be significant barriers to rural ambulatory care appointments. Greater distance from an ED does not improve a clinic’s ability to see semi-urgent appointments. Clinics located near an UC were more likely to grant an appointment than clinics without close alternative outpatient healthcare options.

## INTRODUCTION

Emergency departments (ED) are a routine site of care for patients with conditions that might otherwise be cared for in an ambulatory setting.^1^–^6^ Increased ED visits have resulted in crowding, increased time to treatment, and worse patient outcomes.^7^–^13^ Unreliable access to timely appointments and lack of a primary care physician (PCP) are major causes of patients using the ED rather than alternative sites of care.^4^,^14^–^20^ The increased use of EDs for nonemergency conditions has led to increased healthcare costs for both the patients and hospitals.^21^–^22^

While it is known that scheduling non-emergent visits in an urban setting can be quite challenging, little data has been collected regarding the role of semi-urgent ambulatory care in a non-urban setting.^4^,^14^–^20^ Data regarding barriers to care specifically in rural and suburban areas are lacking.^4^,^14^–^20^,^22^ Trends in barriers to ambulatory care seen in larger cities may not be representative of trends present in other settings.

Up to 80% of established primary care patients are directed to the ED by their PCP upon calling with an exacerbation of symptoms.^14^ The ready availability of the ED to resuscitate and manage patients with multiple complex morbidities has resulted in a shift of the burden of care to settings with easy ED access. It is reasonable to assume that in areas lacking readily available access to EDs or urgent care (UC) clinics, primary care centers would serve as a surrogate for acute care centers and be less likely to refer patients to locations that were significant distances from the original clinic. However, no study to date has examined whether this phenomenon exists.

This study aims to determine the availability of timely access to semi-urgent medical care using sham callers portraying patients traveling outside their usual source of primary care, specifically representing long-distance hikers traveling along the Appalachian Trail (AT). The AT was chosen as a model as it transects rural portions of 14 Eastern states and provides a rational explanation for the transient nature of the sham telephone caller without a local address in a rural setting. We determined the availability of timely access to ambulatory care at primary care facilities and a sham caller’s ability to schedule a semi-urgent appointment in rural areas of the Eastern United States. We evaluated whether calls representing insured patients and uninsured patients able to pay substantial upfront cash fees were more likely to result in a timely appointment than calls representing uninsured patients unable to pay greater than $100. Our second goal was to assess whether a caller representing patients was more or less likely to book a timely appointment at primary care facilities with varying distances from UCs or EDs. We hypothesized that primary care clinics located greater distances from other sources of care would be more amenable to granting an appointment for a non-emergent complaint rather than referring them long distances to UCs or EDs.

## METHODS

### Study Design

We conducted this institutional review board-approved prospective telephone survey of primary healthcare facilities and clinics using a sham telephone caller representing patients attempting to schedule an appointment. The caller was trained to portray clinical vignettes representative of common semi-urgent complaints. For simplicity, any site where outpatient primary care is available was referred to as a clinic. The locations used to draw samples from were private physician offices, hospital and health system clinics, and community clinics.

Clinics were located and mapped using Google Maps (Google, Mountain View, CA) and Google Earth (Google, Mountain View, CA). Of 415 clinics initially selected, 190 were contacted. Those not contacted were unable to be reached due to incorrect or disconnected telephone numbers. Each clinic was contacted by a sham telephone caller trained to portray one of three clinical vignettes designed to represent semi-urgent complaints: an acute musculoskeletal injury, gastrointestinal distress, and a chronic medical condition needing maintenance. Two calls were made to each clinic to assess the effect of insurance and ability to pay cash on a patient’s ability to schedule an appointment representing either an insured or uninsured patient. The sham telephone caller who represented an insured and uninsured patient during these calls was referred to as an insured patient and uninsured patient respectively for brevity.

### Study Setting and Population

We used the AT as a guide for selection of rural and suburban primary care clinics along the Eastern United States. The proximity to the AT provided the sham caller with an explanation for being in the area and needing timely appointments. The clinical scenarios mentioned above were selected because they represent semi-urgent concerns that could have reasonably been experienced by someone traveling the AT and could be evaluated at an ambulatory care facility. We selected care facilities among towns and rural communities within 50 miles of the AT. The clinic location was then mapped to the nearest hospitals with a 24-hour ED and the nearest UC if available. Selected clinics ranged from 0–71 miles from the nearest UC, and 0–42 miles from the nearest ED.

### Study Protocol

A prospective sham patient acting as an AT hiker away from their usual source of primary care called requesting an appointment for same day or next day availability for one of the three following conditions: an acute traumatic event represented by ankle pain and swelling following a stumble, a medical condition of diarrhea persistent for three days, and maintenance of the progression of intermittent asthma requiring increased use of a short acting beta agonist inhaler. The scenario used was randomized for the first call to each clinic; the second call was randomized using the remaining two scenarios not used during the first call.

To eliminate any unforeseen events with the design of the sham calls, a short pilot study was performed to ensure that all necessary data could be reliably and accurately collected using the current scripted vignettes. Three clinics were selected that were within the clinic selection range, but were not included in the selection process for the main study. A trained sham caller portrayed hikers calling with complaints from the AT. No changes to the vignettes or study protocol resulted from the pilot. To minimize variation, one researcher was trained to request the next available appointment slot using one of the three randomized scripted vignettes. Two calls in randomized order, representing either an insured or uninsured patient, were made to each clinic to assess payment ability. If a patient was uninsured and granted an appointment the caller would request the necessary upfront payment required to be seen at each office.

To prevent clinics from becoming suspicious, a minimum of two weeks between each call was allotted, and identifying information of the sham patient was changed. The sham caller gave different identifying information for calls to the same clinic, and scenarios were not repeated at any individual clinic to minimize recognition by clinics’ schedulers. Any available appointments were canceled before the end of each phone call to ensure appointment times were not taken from other potential patients.

Calls were made during standard office hours 8:00am–5:00pm, Monday through Friday. If a clinic’s office hours were different from the standard office hours defined by this study, an additional call was made during the clinic’s regular hours. The time interval from the first call to the repeat call was not included in the calculation of time till next available appointment – the time to next available appointment was started following the second call.

### Measurements

The following data was collected during each call: appointment availability, time between call and scheduled appointment, and required payment method. We assessed timely care as a dichotomous variable - either the patient could successfully schedule an appointment within 48 hours or not. Insurance status of the sham caller was recorded for each call. If the caller was representing an uninsured patient, the required minimum upfront cash payment for the office visit was documented. If the amount of a specific upfront payment was not volunteered, the sham patient inquired about any fees that needed to be paid upfront before or immediately after the office visit. If an amount did need to be paid, the caller would ask if the clinic was supportive of setting up a payment plan, as well as the minimum upfront cost given this option. Almost all sampled clinics had upfront cash fees for uninsured patients, and most commonly a minimum of $100. This dollar amount was used to create two subcategories of uninsured patients: those who hypothetically could pay greater than or equal to $100 upfront, and those unable to pay this upfront fee. The purpose of creating these subgroups was to distinguish between uninsured patients who may or may not be able to afford these large upfront cash payments that are common in rural clinics.

If the facility could not make an appointment, the reason for inability to book an appointment and the location and facility type of other suggested healthcare options was recorded. It was suspected that some facilities might not be able to book appointments for reasons including but not limited to the following: the clinic was no longer accepting new patients, the clinic was not accepting new patients who were not insured, and/or there were no available appointments within a reasonable time period.

We calculated distance to the nearest ED or UC for each clinic using a program written in JAVAscript (Oracle, Redwood City, CA) and using Google Maps Application Programming Interface (Google, Inc. Mountain View, CA). Distances were manually checked to ensure accuracy of the program.

### Data Analysis

We conducted analyses and sample size calculations in R (R Foundation for Statistical Computing, Vienna, Austria).^25^ Each clinic was contacted twice – once representing an insured patient and once representing an uninsured patient. We applied McNemar’s test to discern differences between the insured and uninsured callers. Logistic regression was used to control for distance from clinic to nearest healthcare facility, in order to determine if the scenario used affected a caller’s ability to book an appointment.

To insure an adequate number of clinics were surveyed, sample size calculations were performed. Several assumptions were necessary for sample size calculations with McNemar’s test. An appointment rate of 60% for insured patients was assumed based on previous studies,^15^ with the goal to detect a 20% difference in appointment rate at a significance level of 0.05 with power equal to 0.90. The phi coefficient (ϕ), which quantifies the correlation between the outcome for the insured and uninsured status, was set at ϕ=−0.25, requiring a total of 164 clinics.

## RESULTS

Of 380 requests for appointment made to 190 clinics, only 96 (25.3%) resulted in an appointment booked within the acceptable 48–hour window (Table 1). The chief complaint of the scenario did not influence the ability to schedule an appointment. The odds ratios calculated for a callers with intermittent asthma with mild worsening of symptoms and callers with gastrointestinal distress relative to those with a musculoskeletal complaint are as follows: (OR 0.2, CI [0.03–1.08]) and (OR 0.23, CI [0.05–1.04]). Payer type did appear to affect ability to book an appointment as insured patients and uninsured patients willing to put $100 or greater down as payment were significantly more likely to be accepted (McNemar’s chi-squared=28.6 and p-value <0.001, OR 18 CI 154). Only 47 of the 190 clinics contacted were willing to accept an uninsured patient. Of those 47 clinics, 42 (89.3%) required some form of upfront payment.

Clinics ranged in distance from zero to 35 miles to the nearest UC or ED. Increased distance from the nearest ED had no impact on the likelihood of booking an appointment (OR 1.7 CI [0.4, 11.3]). However, clinics located near an UC were more likely to grant an appointment than clinics without close alternative outpatient healthcare options (OR 2.45, 95% CI [1.19–5.80]).

Table 2 illustrates the results of securing an appointment given the population within a zip code and payer status. Zip code size differs from a town’s population, as some areas have a single zip and others have multiple zip codes within a single town or suburban area. Previous studies have addressed barriers to outpatient care in large urban areas.^15^,^20^ The actual town and city population of this study ranged from 746 to 97,856 based on data from the 2010 census, compared to previous studies whose populations ranged from 420,003 (Atlanta) to 8,175,133 (New York).^15^,^20^

## DISCUSSION

The increased role of the ED in providing non-emergent care has been hypothesized to result from a lack of primary care access, availability of ambulatory care appointments, and general dissatisfaction with non-ED care.^1^–^6^ Given the current rationale for accessing EDs for non-emergent conditions, it would seem reasonable that ambulatory facilities in rural areas, not easily accessible to EDs would fill the role of providing timely care for non-emergent conditions experienced by non-established visiting patients. However, we determined that increased distance from the nearest ED had no impact on the likelihood of booking an appointment. In actuality, clinics located a UC were more likely to grant an appointment than clinics without close alternative outpatient healthcare options (OR 2.45, 95% CI [1.19–5.80]). We believe this paradox may suggest proximity to UCs creates more competition for patients, increasing PCP offices’ willingness to accommodate non-established visiting patients. Alternatively these facilities may be appropriately decreasing the volume burden experienced by PCPs allowing them time to see patients with urgent concerns in a timely manner.

We found only 25% of clinics surveyed were willing to treat a non-established visiting patient within a 48-hour window. Unsurprisingly, insurance status and ability to pay for the office visit upfront was a key predictor of ability to schedule an appointment, with insured patients and patients able to pay an upfront amount greater than $100 significantly more likely to be seen in the designated time frame of 48 hours. Seventy-five percent of rural ambulatory clinics were unwilling to see an uninsured patient not able to pay at least $100 upfront.

The results of this study support a previous study’s findings that insurance status did not seem to make a difference as long as the uninsured patient was willing to pay a significant sum upfront.^15^ Patients unable to pay a substantial amount upfront were also less likely to be able to book an appointment in a timely manner. Both studies found that difficulties booking an appointment were not limited to only the uninsured; one quarter of privately insured patients in this study were unable to book an appointment; one third were unable in large urban areas. One hundred percent of clinics in this study screened callers to determine insurance status, versus 98% found by Asplin et al.^15^

A multitude of reasons may exist for these findings including a clinic not currently accepting new patients, thin operating margins that would not cover the time necessary for establishing a new patient, lack of interest in seeing a patient who would not be establishing care, or inability to accept a certain brand of insurance. These findings may portend future challenges for patients and referring physicians searching for timely access to ambulatory care in rural settings as the insured patient pool is expanded through implementation of the Affordable Care and Patient Protection Act (ACA).

Despite financial disincentives enacted by insurance companies to discourage ED use for non-emergent conditions, it appears that in many cases the ED remains the best option for timely treatment, for both insured and uninsured individuals. This would seem to support literature regarding insured patients’ increased use of the ED.^15^

If the likelihood of insured patients gaining appointments is slim, the likelihood for uninsured patients is much worse. Only 47 of the 190 clinics would accept patients without insurance, and almost 90% of those required upfront payment for services rendered. We believe the ED might remain the safety net for these patients who are outside of their normal healthcare provider range. It is not difficult to draw conclusions regarding future challenges for rural EDs. Even in those states expanding Medicaid through the ACA, receiving care in a non-ED setting can be difficult for patients. It is likely that even rural EDs will suffer from crowding and the associated negative consequences there of.^7^–^13^ It may be safe to assume that decreased Disproportionate Share Reimbursements will further increase the already significant financial strain on rural EDs.^24^

## LIMITATIONS

One limitation of this study is the use of Google search engine for identifying primary care clinics. Of 415 clinics initially identified, only 190 were accessible by telephone number listed in the Google search. This study is also limited by the assumption that the AT is a sufficient surrogate marker for rural settings as well as limiting town/city size. Utilization of Geographical Information Systems to assess urban, suburban, and rural settings could potentially more accurately identify rural settings to target.

The inability for sham callers to provide local addresses could have made clinics less likely to grant appointments due to the decreased likelihood of the patient establishing continued future care. The nature of this study design only allows for conclusions to be drawn for acute new patient appointments for out-of-town patients not planning on establishing care and cannot represent appointment availability for established patients within a primary care practice. Furthermore, focusing on a transient population allowed the sham caller to minimize the amount of deception during the telephone call, eliminating the need to give intent to establish care at the clinic and give a local home address to a clinic scheduler who might be very familiar with the local neighborhoods and their inhabitants.

As our callers were not real patients, social security numbers and specific insurance policy information could not be given out, but this did not inhibit a clinic’s ability to finish with the caller protocol. Clinics were instructed that the patient would call back immediately with this information; however, each appointment was canceled before the end of the phone call, so that this step was no longer necessary.

Cash payments appear to play a significant role in rural primary care visits, as it was commonly requested upfront during identification of uninsured status. We did not explicitly examine this effect other than sham caller’s hypothetical ability to pay the minimum upfront fee. This is of course in stark contrast to ED visits that require no upfront payment. The $100 sum we used as a cutoff for a substantial upfront fee most likely overestimates the amount many uninsured are able to pay for such an office visit. This would suggest our findings might overestimate access to ambulatory care, and these patients may be even less likely to successfully schedule an appointment than our study predicts.

## CONCLUSION

Significant barriers to rural ambulatory care for non-emergent medical conditions exist, resulting in use of EDs for non-emergent care by non-established visiting patients. Not surprisingly, there appears to be a disparity for financially vulnerable populations, leaving those uninsured and unable to pay large upfront fees less likely to be able to acquire timely access to outpatient care for semi-urgent ambulatory concerns. However, barriers to care do not seem to be limited to financially vulnerable populations; it was found that insured patients were also unable to schedule ambulatory care appointments. Primary care centers located greater distances from continuously staffed EDs are not surrogates for ED care. Timely access to outpatient care for urgent ambulatory concerns is necessary to prevent unnecessary ED visits. Barriers to this form of care have the potential to create challenges for both EDs and patients seeking care in facilities other than their established PCP office. Patients with concerns that could not be treated in the outpatient setting will experience increased wait times due to ED crowding. Patients with concerns that could be treated in the outpatient setting will accrue considerable costs that could otherwise be avoided if barriers to this care did not exist. This study helps emphasize that these barriers do in fact exist, but also brings to light the impact UC facilities might have on timely ambulatory healthcare access in rural and suburban areas. Further research evaluating the impact of these sites of care would provide a better understanding if their specific impact on ED utilization in rural and suburban environments.

## Figures and Tables

**Table 1:** Comparison of appointment availability between insured and uninsured patients.

Scenario	Private insurance	Cash payment <$100	Unlimited cash payment
Overall	49/190 (25.8)	15/190 (7.8)	47/190 (24.7)
Gastrointestinal	13/51 (25.5)	7/68 (10.3)	20/68 (29.4)
Musculoskeletal	22/72 (30.6)	4/60 (6.7)	13/60 (21.7)
Chronic disease	14/67 (20.9)	4/62 (6.5)	14/62 (22.6)

**Table 2:** Comparison of appointment availability by clinic zip code population.

Population	Private insurance	Cash payment <$100	Unlimited cash payment
Overall	49/190 (25.8)	15/190 (7.8)	47/190 (24.7)
<10,000	20/84 (23.8)	9/84 (10.7)	19/84 (22.6)
10,000–20,000	15/56 (26.8)	3/56 (5.4)	12/56 (21.4)
20,000–30,000	7/23 (30.4)	1/23 (4.3)	8/23 (34.7)
30,000–40,000	7/26 (26.9)	2/26 (7.7)	8/26 (30.7)
40,000–50,000	0/1 (0.0)	0/1 (0.0)	0/1 (0.0)

## References

[1] Baker DW, Stevens MD, Brook RH (1994). Regular source of ambulatory care and medical care utilization by patients presenting to a public hospital emergency department. JAMA.

[2] Honigman LS, Wiler JL, Rooks S (2013). National study of non-urgent emergency department visits and associated resource utilization. West J Emerg Med.

[3] Afilalo J, Marinovich A, Afilalo M (2004). Nonurgent emergency department patient characteristics and barriers to primary care. Acad Emerg Med.

[4] O’Brien GM, Stein MD, Zierler S (1997). Use of the ED as a regular source of care: associated factors beyond lack of health insurance. Ann Emerg Med.

[5] Northington WE, Brice JH, Zou B (2005). Use of an emergency department by nonurgent patients. Am J Emerg Med.

[6] Ragin DF, Hwang U, Cydulka RK (2005). Reasons for using the emergency department: results of the EMPATH Study. Acad Emerg Med.

[7] Derlet R, Richards J, Kravitz R (2001). Frequent overcrowding in U.S. emergency departments. Acad Emerg Med.

[8] Moskop JC, Sklar DP, Geiderman JM (2009). Emergency department crowding, part 1-concept, causes, and moral consequences. Ann Emerg Med.

[9] Bernstein SL, Aronsky D, Duseja R (2009). The effect of emergency department crowding on clinically oriented outcomes. Acad Emerg Med.

[10] McCarthy ML (2011). Overcrowding in emergency departments and adverse outcomes. BMJ.

[11] Kulstad EB, Sikka R, Sweis RT (2010). ED overcrowding is associated with an increased frequency of medication errors. Am J Emerg Med.

[12] McCarthy ML, Zeger SL, Ding R (2009). Crowding delays treatment and lengthens emergency department length of stay, even among high-acuity patients. Ann Emerg Med.

[13] Sikka R, Mehta S, Kaucky C (2010). ED crowding is associated with an increased time to pneumonia treatment. Am J Emerg Med.

[14] Gonzalez Morganti K, Bauhoff S, Blanchard JC (2013). The evolving role of emergency departments in the United States.

[15] Asplin BR, Rhodes KV, Levy H (2005). Insurance status and access to urgent ambulatory care follow-up appointments. JAMA.

[16] Shippee ND, Shippee TP, Hess EP (2014). An observational study of emergency department utilization among enrollees of Minnesota Health Care Programs: financial and non-financial barriers have different associations. BMC Health Serv Res.

[17] Petersen LA, Burstin HR, O’Neil AC (1998). Nonurgent emergency department visits: the effect of having a regular doctor. Med Care.

[18] Rust G, Ye J, Baltrus P (2008). Practical barriers to timely primary care access: impact on adult use of emergency department services. Arch Intern Med.

[19] Cheung PT, Wiler JL, Ginde AA (2011). Changes in barriers to primary care and emergency department utilization. Arch Intern Med.

[20] Vieth TL, Rhodes KV (2008). Nonprice barriers to ambulatory care after an emergency department visit. Ann Emerg Med.

[21] Krochmal P, Riley TA (1994). Increased health care costs associated with ED overcrowding. Am J Emerg Med.

[22] Foley M, Kifaieh N, Mallon WK (2011). Financial impact of emergency department crowding. West J Emerg Med.

[23] Bennett KJ, Moore CG, Probst JC (2007). Estimating uncompensated care charges at rural hospital emergency departments. J Rural Health.

[24] R Development Core Team (2012). R: A language and environment for statistical computing.

